# Different predictors of neurosurgical intervention and mortality in moderate traumatic brain injury: a nationwide cohort study

**DOI:** 10.1016/j.bas.2026.106173

**Published:** 2026-07-14

**Authors:** Olivia Kiwanuka, Niklas Marklund

**Affiliations:** a Karolinska Institutet, Department of Clinical Science and Education, Södersjukhuset, Stockholm, Sweden; b Stockholm South General Hospital (Södersjukhuset), Department of Surgery, Stockholm, Sweden; c Department of Clinical Sciences Lund, Neurosurgery, Lund University, and Skåne University Hospital, Department of Neurosurgery, Lund, Sweden

**Keywords:** Moderate traumatic brain injury, Neurosurgical intervention, Mortality, Age, Trauma registry

## Abstract

**Introduction:**

Moderate traumatic brain injury (TBI) is heterogeneous. Admission and injury-related variables may predict outcomes differently, but their relative importance is unclear.

**Research question:**

Do admission and injury variables differ in predicting neurosurgical intervention and 30-day mortality in moderate TBI?

**Material and methods:**

In a nationwide retrospective cohort study using the Swedish Trauma Registry (SweTrau; 2018–2023), we included adults with moderate TBI (Glasgow Coma Scale 9–13). The primary outcome was neurosurgical intervention; the secondary outcome was 30-day mortality. For each, two multivariable logistic regression models were fitted: an admission model (demographics, comorbidity, physiology) and an injury model (injury severity and intracranial lesions). Discrimination was assessed using the area under the ROC curve (AUC), compared with DeLong's test, and reassessed within age strata (<65 and ≥ 65 years).

**Results:**

Among 1761 patients, 198 (11%) underwent neurosurgical intervention and 299 (17%) died within 30 days. The injury model discriminated intervention better than the admission model (AUC 0.84 vs 0.67), in both younger and older patients. For mortality the admission model discriminated better overall (0.84 vs 0.78), but only in older patients: among those <65, injury severity discriminated better (0.88 vs 0.72), whereas among those ≥65 the models were equivalent (0.73 vs 0.71). Increasing age was associated with higher mortality but lower odds of intervention.

**Discussion and conclusion:**

In moderate TBI, the information that identifies who needs surgery is not the information that predicts who will survive, and for survival that information itself changes with age. A single severity measure is therefore insufficient.

## Highlights

• In moderate TBI, surgery and 30-day mortality had different predictors.
• Injury variables best predicted neurosurgical intervention, at every age.
• Admission variables predicted mortality mainly in older patients.
• In patients under 65, injury severity predicted mortality better than admission factors.
• Increasing age raised mortality but lowered the odds of neurosurgical intervention.

## Graphical abstract

**Figure:**
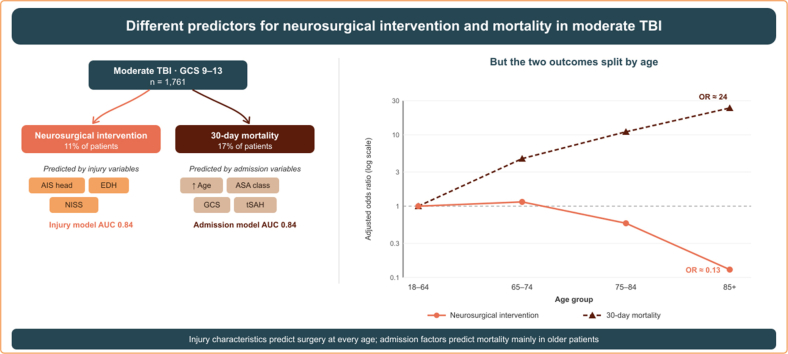

## 1 Introduction

Traumatic brain injury (TBI) is a major cause of death and long-term disability worldwide (Halvachizadeh et al., 2025; van Wessem et al., 2024). Injury severity is commonly classified using the Glasgow Coma Scale (GCS), with Scandinavian guidelines defining mild TBI as GCS 14-15, moderate TBI as 9-13, and severe TBI as 3-8 (Undén et al., 2013). While this classification underpins clinical practice and research, it groups together patients with different clinical trajectories, particularly within the moderate category.

Moderate TBI represents a heterogeneous group positioned between two more clearly defined entities. Severe TBI is typically associated with immediate need for neurosurgical and intensive care management, whereas mild TBI is often managed with observation alone. In contrast, patients with moderate TBI may follow markedly different clinical trajectories. Some recover without complication, while others deteriorate or require urgent neurosurgical intervention (Watanitanon et al., 2018). Identifying those at risk of an unfavorable course remains challenging at the time of presentation.

Predictors of outcome after TBI span both patient-related characteristics and injury-specific features. Admission variables such as age, comorbidity burden, and level of consciousness are consistently linked to mortality and functional prognosis, likely reflecting underlying physiological reserve and vulnerability to secondary insults (Roohollahi et al., 2024; Maas et al., 2022). Radiological findings, on the other hand, reflect structural brain injury more directly, and are central to decisions regarding neurosurgical intervention (Svedung et al., 2023). Despite these differences, these domains are often combined within the same prognostic models, with limited assessment of whether they represent a single dimension of risk or distinct pathways leading to different outcomes.

In clinical practice, early decision-making in moderate TBI involves at least two related but not equivalent questions: which patients are likely to require neurosurgical intervention, and which are at highest risk of death. These outcomes are frequently analyzed together or treated as reflecting overall injury severity, although they may be driven by different types of information. The extent to which admission characteristics and injury morphology differentially relate to these outcomes in moderate TBI remains unclear.

Most prior studies have examined predictors across combined moderate and severe TBI populations or focused on single outcomes. As a result, the relative contributions of admission and injury-related variables to different outcomes within moderate TBI are not well defined.

Using data from the Swedish Trauma Registry (SweTrau), we aimed to identify predictors of neurosurgical intervention in patients with moderate TBI. As a secondary objective, we examined predictors of 30-day mortality in the same population. By evaluating these outcomes in parallel, we assessed whether they are associated with different domains of clinical information.

## 2 Methods

### 2.1 Study design and data source

Data from SweTrau, a nationwide trauma registry that prospectively collects standardized information on trauma patients treated at participating hospitals across Sweden, were used for this retrospective cohort study. The registry records patient characteristics, injury mechanism, injury severity, management, and outcomes according to the Utstein trauma reporting framework (Ringdal et al., 2008).

The study period included trauma activations registered between January 1, 2018, and December 31, 2023. The study was conducted and reported in accordance with the STROBE (Strengthening the Reporting of Observational Studies in Epidemiology) guidelines.

### 2.2 Study population

All adult trauma patients recorded in SweTrau during the study period were screened for eligibility. To avoid duplicate registrations of the same injury event, only the index event per patient was included.

Patients younger than 18 years were excluded. Traumatic brain injury was defined by the presence of an ICD-10 diagnosis code in the S06 range (Supplementary Table 1). Patients with missing emergency department (ED) GCS were excluded.

The study population was limited to patients with moderate TBI, defined as an ED GCS score between 9 and 13 in accordance with Swedish national trauma guidelines. ED GCS was used rather than prehospital GCS as it represents the first standardized neurological assessment performed at the receiving hospital.

Patients transferred from hospitals that also report to SweTrau were excluded to avoid duplicate representation of the same injury episode. Transfers from hospitals not reporting to SweTrau were retained because the initial presentation would otherwise not be captured in the registry.

### 2.3 Outcome definitions

The primary outcome was neurosurgical intervention during the index admission. Neurosurgical intervention was defined using procedure codes from the Swedish Classification of Health Interventions (Klassifikation av vårdåtgärder, KVÅ), the national procedure coding system used in Swedish healthcare. Procedures consistent with active neurosurgical management of traumatic intracranial injury, including craniotomy, evacuation of intracranial hematoma, decompressive procedures, and insertion of an intracranial pressure monitor or external ventricular drain, were identified using predefined KVÅ codes recorded in SweTrau (Supplementary Table 2). Intracranial pressure monitoring and drainage were included because they are invasive procedures that both guide and constitute active neurosurgical management, rather than only surgical evacuation of a lesion.

The secondary outcome was 30-day mortality, defined as death occurring within 30 days of the recorded date of injury in SweTrau.

### 2.4 Predictor variables

Candidate predictors were grouped into two predefined domains reflecting different stages of clinical assessment.

#### 2.4.1 Admission variables

Admission variables represented information available at initial evaluation prior to radiological assessment. These included age group (18-64, 65-74, 75-84, and ≥85 years), sex, American Society of Anesthesiologists (ASA) physical status classification (collapsed as ASA 1, 2, 3, and ≥4), ED GCS grouped as 9–11 and 12–13, and hypotension defined as ED systolic blood pressure <90 mmHg.

#### 2.4.2 Injury severity

Injury severity variables were derived from injury scoring. Abbreviated Injury Score (AIS) is an anatomical trauma score primarily based on radiological findings, mainly on CT (Loftis et al., 2018). Injuries are graded on a scale from 1 (minor) to 6 (maximal, fatal) and are categorized into eight anatomical regions: head, face, neck, thorax, abdomen, spine, upper extremities, lower extremities, and external. The New Injury Severity Score (NISS) is an anatomical injury severity measure calculated as the sum of the squares of the three most severe AIS scores, regardless of body region (Osler et al., 1997). We included AIS head score grouped as 0-2, 3-4, and ≥5, NISS (continuous), polytrauma defined as AIS ≥3 in at least two anatomical regions, and the presence of specific intracranial lesions: epidural hematoma (EDH), subdural hematoma (SDH), traumatic subarachnoid hemorrhage (tSAH), contusion, and intracerebral hemorrhage (ICH) derived from AIS scoring (Supplementary Table 3). The total number of distinct intracranial lesion types was also recorded as an ordinal variable (0, 1, or ≥2). AIS region scores were derived from individual AIS codes recorded in SweTrau, with the highest severity score per anatomical region used for all calculations.

### 2.5 Statistical analysis

Two separate multivariable models were specified a priori to reflect clinically distinct domains of information available at different stages of assessment, rather than to maximize predictive performance within a single model.

All analyses were conducted in R (version 4.5). Continuous variables are reported as median with interquartile range (IQR) and categorical variables as frequency and percentage. Differences between groups were assessed using the Wilcoxon rank-sum test for continuous variables and Pearson's chi-squared or Fisher's exact test for categorical variables, as appropriate. Statistical significance was set at α = 0.05; all tests were two-tailed.

Univariable logistic regression was performed for each candidate predictor against each outcome separately, yielding unadjusted odds ratios (OR) with 95% confidence intervals and p-values.

For each outcome, two pre-specified multivariable logistic regression models were fitted, reflecting the two clinical domains described above. Model 1 (the admission model) included age group, sex, ASA class, ED GCS group, and hypotension. Model 2 (the injury characterization model) included AIS head score, NISS, polytrauma, EDH, SDH, tSAH, contusion, ICH, and total number of intracranial lesions. All multivariable models used simultaneous entry of pre-specified predictors without stepwise selection. For each outcome, the admission and injury models were fitted on the same complete-case sample so that their discrimination could be compared on an identical set of patients. Multicollinearity was assessed using variance inflation factors (VIF). No evidence of problematic collinearity or coefficient instability was observed, although some expected overlap was present among injury severity variables (Supplementary Table 3, Supplementary Fig. 1).

Model discrimination was assessed using the area under the receiver operating characteristic curve (AUC) with 95% confidence intervals, and the admission and injury models were compared for each outcome using DeLong's test. Model calibration was assessed visually using calibration plots based on deciles of predicted risk (Supplementary Fig. 2). Adjusted odds ratios for age from the admission models were displayed for both outcomes to illustrate their divergent associations.

To assess whether the outcome-specific model performance and the divergent age associations were attributable to age itself we additionally performed a sensitivity analysis stratified by age (<65 and ≥ 65 years). Within each stratum, both models were refitted and their discrimination compared using DeLong's test. In the <65 stratum, age group comprised a single category and was omitted from the admission model. Because some subgroups, particularly deaths among patients aged <65, contained few events, stratified AUCs are reported as apparent estimates and interpreted as indicating the direction of between-model differences rather than precise values.

Missing data were minimal across variables (<6%) and were not concentrated in key predictors, therefore complete-case analysis was performed.

### 2.6 Ethical considerations

SweTrau data are collected under national registry regulations with ethical approval for registry-based research (DNR, 2025-02316-01). The study was conducted in accordance with GDPR and Swedish national data protection legislation. No individual patient contact was required.

## 3 Results

### 3.1 Study cohort

**Fig. 1:**
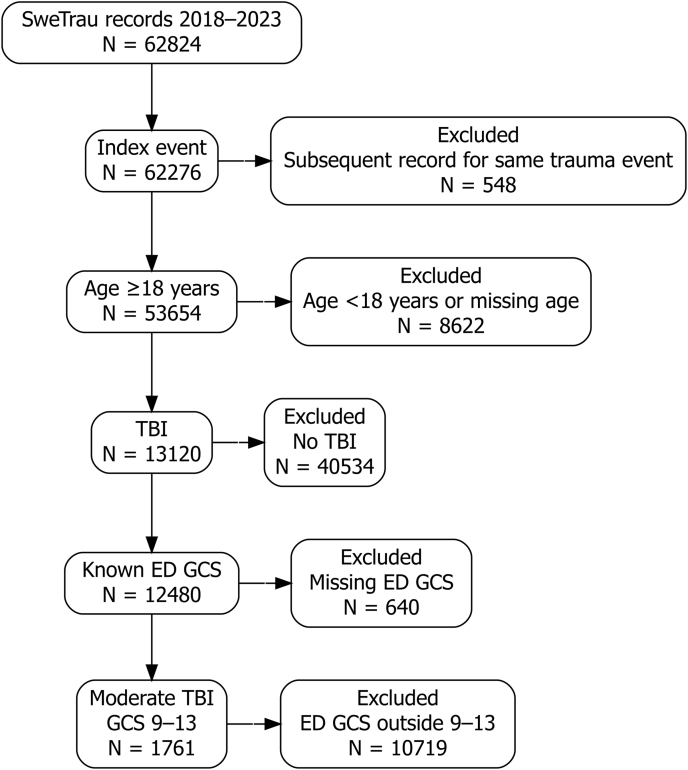
Study flowchart Flowchart of patient selection from the Swedish Trauma Registry (SweTrau) between 2018 and 2023. Patients aged ≥18 years with traumatic brain injury and available emergency department Glasgow Coma Scale (GCS) scores were included. Moderate traumatic brain injury was defined as GCS 9–13. Abbreviations: TBI = traumatic brain injury; GCS = Glasgow Coma Scale; ED = emergency department.

A total of 1761 patients with moderate traumatic brain injury (ED GCS 9–13) were included in the final analytic cohort (Fig. 1). Of these, 198 patients (11.2%) underwent neurosurgical intervention, and 299 patients (17.0%) died within 30 days of injury. Missingness was low across all variables (Supplementary Table 4).

### 3.2 Baseline characteristics

Baseline characteristics stratified by neurosurgical intervention are presented in Table 1. Patients who underwent neurosurgical intervention were younger than those who did not (median age 59 vs 64 years, p < 0.001), while sex distribution and ASA class were similar between groups. Patients in the neurosurgical group more frequently presented with GCS 9–11, had higher injury severity (median NISS 34 vs 22, p < 0.001), and more often had AIS head score 5. Intracranial lesions were more common among patients undergoing neurosurgical intervention. Subdural hematoma and epidural hematoma were more frequent, and contusions were also more common. Isolated subdural hematoma was more frequent in the neurosurgical group, whereas isolated traumatic subarachnoid hemorrhage was less common. Polytrauma was also more frequent in this group (22% vs 15%, p = 0.020). Thirty-day mortality did not differ significantly between groups.

**Table 1:** Baseline characteristics of moderate TBI patients stratified by neurosurgical intervention.

Variable	No neurosurgery N = 1563^a^ (89%)	Neurosurgery N = 198^a^ (11%)	p-value^2^
**Age, years**	64 (43, 79)	59 (38, 71)	<0.001*
**Sex (male)**	1043 (67%)	135 (68%)	0.7
**ASA class**			0.2
ASA 1	487 (32%)	64 (33%)	
ASA 2	495 (32%)	70 (36%)	
ASA 3	528 (34%)	60 (31%)	
ASA 4+	36 (2.3%)	1 (0.5%)	
**Mechanism of injury**			0.013*
Traffic	428 (27%)	45 (23%)	
Falls, high energy	407 (26%)	57 (29%)	
Falls, low energy	577 (37%)	62 (31%)	
Penetrating	9 (0.6%)	2 (1.0%)	
Other	142 (9.1%)	32 (16%)	
**ED GCS**			<0.001*
9-11	540 (35%)	102 (52%)	
12-13	1023 (65%)	96 (48%)	
**ED systolic blood pressure, mmHg**	138 (120, 156)	140 (125, 160)	0.11
**Hypotension (SBP <90)**	30 (2.0%)	3 (1.7%)	>0.9
**AIS head**			<0.001*
0	3 (0.2%)	0 (0%)	
1	116 (7.4%)	0 (0%)	
2	378 (24%)	2 (1.0%)	
3	564 (36%)	33 (17%)	
4	272 (17%)	49 (25%)	
5	230 (15%)	114 (58%)	
**NISS**	22 (10, 29)	34 (27, 43)	<0.001*
**Isolated TBI**	758 (48%)	98 (49%)	0.8
**Polytrauma**	239 (15%)	43 (22%)	0.020*
**EDH, total**	106 (6.8%)	50 (25%)	<0.001*
Isolated EDH	25 (1.6%)	3 (1.5%)	>0.9
**SDH, total**	698 (45%)	148 (75%)	<0.001*
Isolated SDH	164 (10%)	33 (17%)	0.012*
**tSAH, total**	793 (51%)	122 (62%)	0.004*
Isolated tSAH	215 (14%)	5 (2.5%)	<0.001*
**Contusion, total**	447 (29%)	94 (47%)	<0.001*
Isolated contusion	66 (4.2%)	4 (2.0%)	0.2
**ICH, total**	117 (7.5%)	21 (11%)	0.12
Isolated ICH	32 (2.0%)	1 (0.5%)	0.2
**30-day mortality**	273 (17%)	26 (13%)	0.13

Table 1. Baseline characteristics of moderate TBI patients stratified by neurosurgical intervention Baseline characteristics of patients with moderate traumatic brain injury stratified by neurosurgical intervention.

^2^Wilcoxon rank sum test; Pearson's Chi-squared test; Fisher's exact test. Statistically significant values (p < 0.05) are marked with an asterisk.

Abbreviations: NISS = New Injury Severity Score; AIS = Abbreviated Injury Scale; EDH = epidural hematoma; SDH = subdural hematoma; tSAH = traumatic subarachnoid hemorrhage; ICH = intracerebral hemorrhage.

a Median (Q1, Q3); n (%).

Baseline characteristics stratified by 30-day mortality are presented in Table 2. Patients who died were older than survivors (median age 82 vs 58 years, p < 0.001), more often female, and had higher ASA class. Non-survivors more frequently presented with GCS 9–11 and had higher injury severity, including a greater proportion with AIS head score 5. Intracranial hemorrhagic lesions were more common among patients who died, particularly subdural hematoma and traumatic subarachnoid hemorrhage. Isolated subdural hematoma was more frequent among non-survivors, while isolated contusion was less common. The proportion undergoing neurosurgical intervention did not differ significantly between survivors and non-survivors.

**Table 2:** Baseline characteristics of moderate TBI patients stratified by 30-day mortality.

Variable	Alive N = 1462^a^ (83%)	Deceased N = 299^a^ (17%)	p-value^2^
**Age, years**	58 (37, 73)	82 (75, 88)	<0.001*
**Sex (male)**	1003 (69%)	175 (59%)	<0.001*
**ASA class**			<0.001*
ASA 1	532 (37%)	19 (6.4%)	
ASA 2	492 (34%)	73 (24%)	
ASA 3	405 (28%)	183 (61%)	
ASA 4+	14 (1.0%)	23 (7.7%)	
**Mechanism of injury**			
Traffic	447 (31%)	26 (8.7%)	
Falls, high energy	386 (26%)	78 (26%)	
Falls, low energy	455 (31%)	184 (62%)	
Penetrating	11 (0.8%)	0 (0%)	
Other	163 (11%)	11 (3.7%)	
**ED GCS**			<0.001*
9-11	503 (34%)	139 (46%)	
12-13	959 (66%)	160 (54%)	
**ED systolic blood pressure, mmHg**	136 (120, 153)	150 (130, 170)	<0.001*
**Hypotension (SBP <90)**	25 (1.8%)	8 (2.8%)	0.3
**AIS head**			<0.001*
0	2 (0.1%)	1 (0.3%)	
1	114 (7.8%)	2 (0.7%)	
2	352 (24%)	28 (9.4%)	
3	520 (36%)	77 (26%)	
4	261 (18%)	60 (20%)	
5	213 (15%)	131 (44%)	
**NISS**	22 (10, 29)	27 (22, 38)	<0.001*
**Isolated TBI**	674 (46%)	182 (61%)	<0.001*
**Polytrauma**	235 (16%)	47 (16%)	0.9
**EDH, total**	135 (9.2%)	21 (7.0%)	0.3
Isolated EDH	27 (1.8%)	1 (0.3%)	0.072
**SDH, total**	643 (44%)	203 (68%)	<0.001*
Isolated SDH	150 (10%)	47 (16%)	0.009*
**tSAH, total**	717 (49%)	198 (66%)	<0.001*
Isolated tSAH	185 (13%)	35 (12%)	0.7
**Contusion, total**	461 (32%)	80 (27%)	0.11
Isolated contusion	65 (4.4%)	5 (1.7%)	0.022*
**ICH, total**	101 (6.9%)	37 (12%)	0.003*
Isolated ICH	25 (1.7%)	8 (2.7%)	0.2
**Neurosurgical intervention**			0.13
No neurosurgery	1290 (88%)	273 (91%)	
Neurosurgery	172 (12%)	26 (8.7%)	

Table 2. Baseline characteristics of moderate TBI patients stratified by 30-day mortality. Baseline characteristics of patients with moderate traumatic brain injury stratified by 30-day mortality.

^2^Wilcoxon rank sum test; Pearson's Chi-squared test; Fisher's exact test. Statistically significant values (p < 0.05) are marked with an asterisk.

Abbreviations: NISS = New Injury Severity Score; AIS = Abbreviated Injury Scale; EDH = epidural hematoma; SDH = subdural hematoma; tSAH = traumatic subarachnoid hemorrhage; ICH = intracerebral hemorrhage.

a Median (Q1, Q3); n (%).

### 3.3 Primary outcome: neurosurgical intervention

Multivariable associations with neurosurgical intervention are shown in the neurosurgical intervention columns of Table 3 (admission model) and Table 4 (injury model). Univariable associations are provided in Supplementary Table 5.

**Table 3:** Admission model: multivariable associations with neurosurgical intervention and 30-day mortality.

Variable	Neurosurgical intervention		30-day mortality	
	OR	p-value	OR	p-value
**Sex**				
F	—		—	
M	1.06 (0.75, 1.51)	0.7	0.93 (0.68, 1.27)	0.7
**Age group**				
18-64	—		—	
65-74	1.14 (0.75, 1.72)	0.5	4.59 (2.69, 8.01)	<0.001*
75-84	0.58 (0.35, 0.95)	0.035*	11.0 (6.66, 18.7)	<0.001*
85+	0.13 (0.05, 0.32)	<0.001*	23.8 (14.1, 41.5)	<0.001*
**ASA class**				
ASA 1	—		—	
ASA 2	1.18 (0.80, 1.75)	0.4	1.82 (1.03, 3.32)	0.045*
ASA 3	1.29 (0.82, 2.01)	0.3	2.73 (1.57, 4.96)	<0.001*
ASA 4+	0.30 (0.02, 1.50)	0.2	12.5 (5.02, 32.4)	<0.001*
**ED GCS**				
12-13	—		—	
9-11	2.23 (1.63, 3.06)	<0.001*	1.75 (1.29, 2.37)	<0.001*
**Hypotension (SBP <90)**				
No	—		—	
Yes	0.49 (0.08, 1.70)	0.3	1.30 (0.47, 3.40)	0.6

Table 3. Multivariable logistic regression of admission variables associated with neurosurgical intervention and 30-day mortality.

Abbreviations: OR = odds ratio; CI = confidence interval. Statistically significant values (p < 0.05) are marked with an asterisk.

**Table 4:** Injury model: multivariable associations with neurosurgical intervention and 30-day mortality.

Variable	Neurosurgical intervention		30-day mortality	
	OR	p-value	OR	p-value
**NISS**	1.04 (1.02, 1.06)	<0.001*	1.03 (1.01, 1.04)	0.003*
**Polytrauma**				
No	—		—	
Yes	1.02 (0.64, 1.61)	>0.9	0.63 (0.41, 0.96)	0.033*
**AIS head**				
0-2	—		—	
3-4	5.23 (1.45, 33.6)	0.030*	2.53 (1.48, 4.41)	<0.001*
5+	14.1 (3.64, 94.4)	<0.001*	6.61 (3.33, 13.3)	<0.001*
**EDH**				
No	—		—	
Yes	1.96 (1.17, 3.24)	0.010*	0.39 (0.21, 0.69)	0.002*
**SDH**				
No	—		—	
Yes	1.12 (0.69, 1.84)	0.7	1.43 (0.89, 2.32)	0.14
**ICH**				
No	—		—	
Yes	0.94 (0.50, 1.71)	0.8	1.10 (0.64, 1.88)	0.7
**tSAH**				
No	—		—	
Yes	0.78 (0.48, 1.29)	0.3	2.68 (1.61, 4.55)	<0.001*
**Contusion**				
No	—		—	
Yes	1.02 (0.66, 1.59)	>0.9	0.49 (0.33, 0.73)	<0.001*
**Intracranial lesions, n**				
0	—		—	
1	3.89 (1.22, 17.6)	0.040*	0.82 (0.43, 1.56)	0.5
2+	5.57 (1.38, 29.3)	0.024*	0.50 (0.17, 1.45)	0.2

Table 4. Multivariable logistic regression of injury severity variables associated with neurosurgical intervention and 30-day mortality.

Abbreviations: OR = odds ratio; CI = confidence interval; NISS = New Injury Severity Score; AIS = Abbreviated Injury Scale; EDH = epidural hematoma; SDH = subdural hematoma; tSAH = traumatic subarachnoid hemorrhage; ICH = intracerebral hemorrhage. Statistically significant values (p < 0.05) are marked with an asterisk.

In the admission model (Table 3; Fig. 3A), GCS 9–11 was associated with neurosurgical intervention (OR 2.23, 95% CI 1.63–3.06, p < 0.001). Increasing age was associated with lower adjusted odds of intervention, with OR 0.58 (95% CI 0.35–0.95, p = 0.035) for age 75–84 years and OR 0.13 (95% CI 0.05–0.32, p < 0.001) for age ≥85 years, compared with age 18–64 years. Sex, ASA class, and hypotension were not associated with neurosurgical intervention.

In the injury model (Table 4; Fig. 3C), higher NISS (OR 1.04 per point, 95% CI 1.02–1.06) and higher AIS head scores (AIS head 3–4, OR 5.23; AIS head 5+, OR 14.1) were associated with neurosurgical intervention, as was epidural hematoma (OR 1.96, 95% CI 1.17–3.24, p = 0.010). The number of intracranial lesions reached statistical significance but with wide confidence intervals, reflecting the small number of operated patients without a recorded lesion; other individual lesion types and polytrauma were not independently associated after adjustment.

**Fig. 2:**
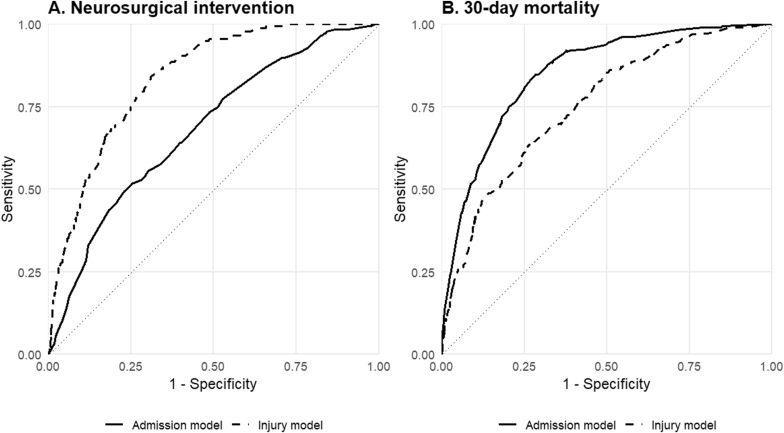
Receiver operating characteristic curves for prediction models Receiver operating characteristic curves for logistic regression models predicting neurosurgical intervention (A) and 30-day mortality (B) based on admission variables and injury severity variables. Abbreviations: ROC = receiver operating characteristic; AUC = area under the receiver operating characteristic curve.

The injury model showed higher discrimination for neurosurgical intervention than the admission model (AUC 0.84 vs 0.67; Table 5, Fig. 2A). Calibration was acceptable across models (Supplementary Fig. 2).

**Table 5:** Model discrimination for neurosurgical intervention and 30-day mortality, overall and by age stratum.

Outcome	Population	Admission model AUC (95% CI)	Injury model AUC (95% CI)	DeLong p
Neurosurgical intervention	Whole cohort	0.67 (0.63-0.71)	0.84 (0.81-0.86)	<0.001*
	Age <65	0.64 (0.58-0.70)	0.87 (0.83-0.90)	<0.001*
	Age ≥65	0.72 (0.66-0.78)	0.83 (0.79-0.87)	0.001*
30-day mortality	Whole cohort	0.84 (0.82-0.87)	0.78 (0.75-0.81)	0.001*
	Age <65	0.72 (0.63-0.82)	0.88 (0.81-0.95)	<0.001*
	Age ≥65	0.73 (0.69-0.77)	0.71 (0.68-0.75)	0.592

AUC, area under the receiver operating characteristic curve. The DeLong p-value compares the admission and injury models within each population. In the age <65 stratum the admission model excludes age group, which has a single level; see Methods. Subgroup estimates are apparent (in-sample) values and, where events are few (30-day mortality in patients <65), indicate the direction of the difference rather than a precise estimate. Statistically significant values (p < 0.05) are marked with an asterisk.

**Fig. 3:**
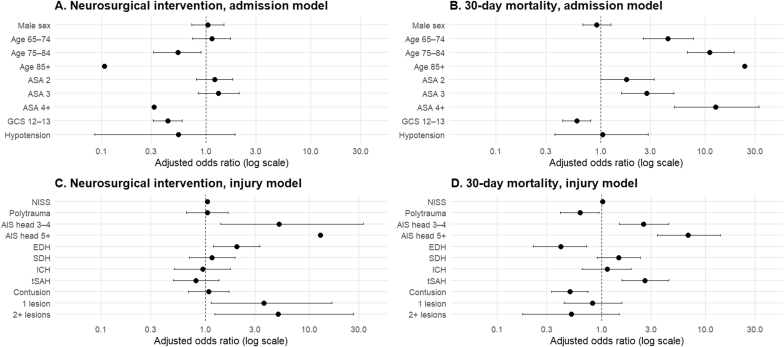
Forest plots of multivariable admission models Forest plots showing adjusted odds ratios with 95% confidence intervals from multivariable logistic regression models. Panels A and B show results from the admission model for neurosurgical intervention (A) and 30-day mortality (B). Panels C and D show results from the injury model for neurosurgical intervention (C) and 30-day mortality (D). Abbreviations: OR = odds ratio; CI = confidence interval; ASA = American Society of Anesthesiologists; GCS = Glasgow Coma Scale; NISS = New Injury Severity Score; AIS = Abbreviated Injury Scale; EDH = epidural hematoma; SDH = subdural hematoma; tSAH = traumatic subarachnoid hemorrhage; ICH = intracerebral hemorrhage.

### 3.4 Secondary outcome: 30-day mortality

Multivariable associations with 30-day mortality are shown in the 30-day mortality columns of Table 3 (admission model) and Table 4 (injury model). Univariable associations are provided in Supplementary Table 6.

In the admission model (Table 3; Fig. 3B), age was associated with mortality in a stepwise manner, from OR 4.59 (95% CI 2.69–8.01) at age 65–74 to OR 23.8 (95% CI 14.1–41.5) at age ≥85, relative to age 18–64 (all p < 0.001). Higher ASA class was also associated with mortality (ASA 4+, OR 12.5, 95% CI 5.02–32.4, p < 0.001), as was GCS 9–11 (OR 1.75, 95% CI 1.29–2.37, p < 0.001). Sex and hypotension were not associated with mortality.

In the injury model (Table 4; Fig. 3D), higher NISS and higher AIS head scores were associated with mortality (AIS head 5+, OR 6.61, 95% CI 3.33–13.3). Traumatic subarachnoid hemorrhage was associated with increased odds of death (OR 2.68, 95% CI 1.61–4.55), whereas epidural hematoma (OR 0.39), contusion (OR 0.49), and polytrauma (OR 0.63) were associated with lower odds.

The admission model showed higher discrimination for 30-day mortality than the injury model (AUC 0.84 vs 0.78; Table 5, Fig. 2B).

### 3.5 Age and outcomes

**Fig. 4:**
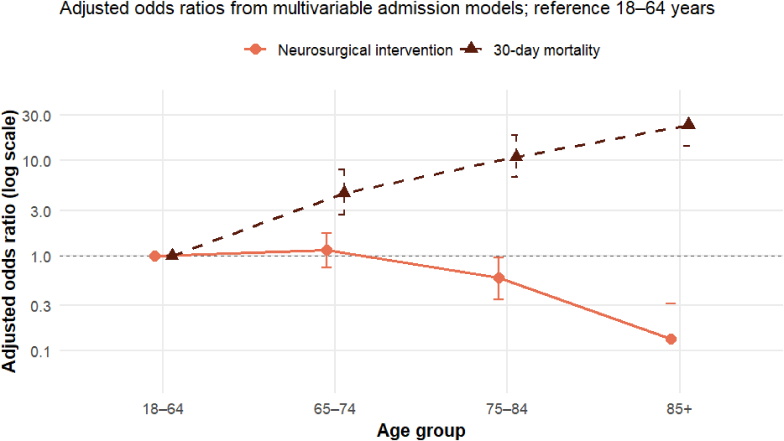
Diverging associations between age and outcomes Adjusted odds ratios for age group from the multivariable admission models, with 18–64 years as the reference. Circles show neurosurgical intervention; triangles show 30-day mortality. With increasing age, the adjusted odds of death rise while the adjusted odds of neurosurgical intervention fall, the two associations diverging across age groups. Error bars are 95% confidence intervals; the odds-ratio axis is on a logarithmic scale. Abbreviations: OR = odds ratio; CI = confidence interval.

Adjusted odds ratios for age group in the multivariable admission models are shown in Fig. 4: with increasing age, the odds of 30-day mortality rose while the odds of neurosurgical intervention fell, the two associations diverging across age groups. To examine whether this divergence, and the outcome-specific model performance, depended on age, both models were refitted within age strata (<65 and ≥ 65 years); discrimination is reported in Table 5 and the corresponding ROC curves in Supplementary Fig. 3.

For neurosurgical intervention, the injury model out-discriminated the admission model in both strata (AUC 0.87 vs 0.64 in patients <65, p < 0.001; 0.83 vs 0.72 in those ≥65, p = 0.001), and the lower adjusted odds of intervention with advancing age were confined to the ≥65 stratum. For 30-day mortality, the pattern differed by age: among patients <65 the injury model discriminated death better than the admission model (0.88 vs 0.72, p < 0.001), whereas among those ≥65 the two models were equivalent (0.73 vs 0.71, p = 0.59). The whole-cohort advantage of the admission model for mortality was therefore not present within age strata. These stratified estimates are apparent values and, in the smaller subgroups, particularly mortality in patients <65, where deaths were few, should be interpreted as indicating the direction of the difference rather than precise estimates.

## 4 Discussion

Early care in moderate TBI turns on two largely separate questions: whether the patient needs a neurosurgical procedure, and whether they will survive. In this nationwide cohort of 1761 adults, these questions were answered by different information. The need for intervention was best discriminated by intracranial lesion characteristics, and mortality by characteristics available at admission, such that the model built for one outcome discriminated the other less well. This separation was not uniform across the cohort: injury characteristics predicted operative need at every age, whereas the admission variables' advantage for predicting mortality was confined to older patients and was carried largely by age itself. The information that identifies who needs surgery is therefore stable across the age range, while the information that predicts survival shifts with it.

Moderate TBI is recognized as a heterogeneous group, with trajectories ranging from uncomplicated recovery to rapid deterioration (Watanitanon et al., 2018; Lund et al., 2016). Our results extend this by showing that the variables most strongly associated with outcomes differ within the same population, and that for mortality the relevant predictors themselves shift with age.

We separated admission and injury-related variables a priori to reflect how patients are assessed in practice. Admission variables represent characteristics available at presentation, including demographics, comorbidity, and physiology, whereas injury variables are based on anatomical injury severity and intracranial findings. Although related, their contribution to outcome differed, and this pattern was consistent across both regression analyses and model performance.

The association between admission variables and mortality is consistent with previous work, particularly regarding age (Watanitanon et al., 2018; Aries et al., 2024; Demetriades et al., 2004), and ASA class (Kiwanuka et al., 2025, 2026). Age showed opposite associations with the two outcomes, increasing the odds of death while decreasing the likelihood of neurosurgical intervention. Stratifying at 65 years located where each of these effects resides. For mortality, the admission model's advantage was an older-patient phenomenon: among patients <65, mortality was better discriminated by injury severity than by admission variables (area under the curve 0.88 versus 0.72), whereas among those ≥65 the two performed equivalently. Admission variables therefore appear better for mortality only because most patients in this cohort are older and age strongly predicts death; within a single age group this apparent advantage disappears. Within the admission model, this signal was carried chiefly by age itself, with comorbidity (ASA class) contributing independently and reduced GCS retaining an independent association with death (odds ratio 1.75; Table 3); acute physiological derangement such as hypotension added little in this cohort, in which few patients were hemodynamically unstable. For neurosurgical intervention, by contrast, the injury model out-discriminated the admission model in both strata, indicating that operative need is read from the injury regardless of age.

The declining likelihood of surgery with advancing age was confined to older patients, and its interpretation is genuinely open. A reduced operative rate in a 75-year-old relative to a 45-year-old with comparable imaging may reflect appropriate patient selection, a less favourable balance of operative risk and benefit, or age-based limitation of care. These possibilities cannot be distinguished in registry data that do not record treatment-limitation decisions. Previous studies have also shown that age modifies the prognostic value of both GCS and injury severity (Demetriades et al., 2004), complicating the interpretation of associations with intervention.

One consequence deserves explicit statement, because it is easily obscured when outcomes are analyzed across all ages together. Among patients aged ≥65, a small set of characteristics available at the bedside, principally age and comorbidity, discriminated 30-day mortality as well as the full characterization of the intracranial lesions (0.73 vs 0.71, p = 0.59). In older patients with moderate TBI, knowing the patient's age and baseline health conveyed as much prognostic information about death as characterizing the intracranial lesions and overall injury severity combined, with age the single strongest correlate of mortality in this group. The prognostic weight of age and comorbidity in older trauma patients is well recognized (Aries et al., 2024; Demetriades et al., 2004; Kiwanuka et al., 2026); their parity with, rather than subordination to, injury severity in moderate TBI has been less clearly stated. The practical corollary is that injury-focused triage and anatomical severity scores, which perform well for identifying operative need, may under-weight the host factors that most strongly determine survival in the older patients who now make up the majority of this population.

A plausible interpretation of this age-dependence is physiological reserve. The decision to operate is driven by the intracranial lesion despite the patient's age and comorbidity: the injury variables predicted intervention equally well in younger and older patients, whereas age and comorbidity did not. Survival, by contrast, depends on whether the patient can withstand the injury. Younger patients tolerate insults that prove fatal in older patients, so among the young it is largely the anatomical severity of the injury that separates survivors from those who die, which is why injury severity dominated mortality prediction in this group. In older patients, host factors such as age, comorbidity, and the diminished reserve that accompanies them, reach a prognostic weight comparable to the injury itself, so the two domains converge rather than injury becoming unimportant. This mechanism is interpretive and cannot be confirmed with the present data, but it accounts for the age-dependence of the mortality findings without appealing to treatment limitation alone.

Injury-related variables dominated in the prediction of neurosurgical intervention. Higher injury severity and the presence of intracranial lesions were strongly associated with surgery, as expected given that these factors inform operative decisions (Aries et al., 2024). Epidural hematoma was associated with increased likelihood of intervention and lower mortality, consistent with its role as a surgically treatable lesion. That GCS predicted intervention across age strata, while the relationship between GCS and head AIS remained weak, further highlights that clinical and anatomical measures provide different information (Demetriades et al., 2004).

Traumatic subarachnoid hemorrhage showed a different pattern. It was less common among patients undergoing neurosurgical intervention but more common among those who died. This is consistent with prior studies demonstrating low rates of clinical deterioration and minimal need for operative management in patients with isolated tSAH (Bellen et al., 2024). This aligns with its association with overall injury burden rather than serving as a direct indication for surgery. Previous studies suggest that associated injuries and hemorrhage characteristics are more important than the presence of tSAH alone, which fits with our findings (Wong et al., 2011; Wu et al., 2020).

Global injury severity also played a role. Injury severity scores such as ISS and NISS have been shown to improve mortality prediction, with NISS capturing the three most severe injuries regardless of body region (Osler et al., 1997), but these measures do not isolate brain-specific pathology. Extracranial injuries have also been linked to worse outcomes and increased resource use in moderate TBI (Watanitanon et al., 2018; Lund et al., 2016), and may contribute to the associations with mortality observed here. In our data, injury severity was associated with both outcomes, but with different strengths of association. This may partly explain why models aligned with specific outcomes performed differently.

These findings have implications for early management. In moderate TBI, clinicians must consider both overall risk and the likelihood of requiring intervention, and our results indicate that no single variable or risk model serves both questions equally well: a tool calibrated to predict mortality will not rank operative need well, and vice versa. Which admission information is most informative for mortality depends on age, and some host factors that are decisive in the elderly add little in the young. Preventing secondary insults remains central to care (Grevfors et al., 2021; Moon and Park, 2026), while studies of interhospital transfer suggest that delays alone do not necessarily worsen outcomes once patient characteristics are accounted for (Grevfors et al., 2021; Moon and Park, 2026; Rahim et al., 2022).

Several limitations should be considered when interpreting these findings. First, moderate TBI is defined using GCS, which is an imperfect measure and may be affected by factors such as intoxication and interobserver variability (Demetriades et al., 2004; Eghzawi et al., 2024). Second, the outcomes examined represent observed clinical events rather than underlying need. Neurosurgical intervention was defined as performed intervention and is therefore influenced by clinical decision-making, treatment limitations, and healthcare system factors. This may be particularly relevant in older or frail patients, where intervention is more likely to be withheld. Similarly, mortality was analyzed without information on cause of death, limiting the ability to distinguish deaths directly attributable to brain injury from those related to comorbidities or treatment decisions. Prior studies have highlighted the influence of triage, transfer decisions, and patient selection on observed outcomes (Grevfors et al., 2021; Moon and Park, 2026; Rahim et al., 2022).

In this cohort, neurosurgical intervention and mortality were associated with different predictors, injury characteristics with intervention, robustly across age, and admission characteristics with mortality, an association concentrated in older patients and driven largely by age and comorbidity. Recognizing that the drivers of these two outcomes differ, and that the predictors of survival themselves vary with age, may help interpret risk and inform patient selection in moderate TBI. Two questions follow from this work. First, whether the same split holds in mild and severe TBI, which we did not study here. Second, whether other outcomes, such as functional recovery and length of stay, show similar patterns. Both are left for future work.

## 5 Conclusions

In this nationwide cohort of adults with moderate traumatic brain injury, the need for neurosurgical intervention was associated with the lesion characteristics, whereas mortality was associated with admission characteristics, an association concentrated in older patients, in whom a small set of host factors predicted death as accurately as the full injury characterization. A single severity measure is therefore insufficient in moderate TBI: the information that identifies who needs surgery is not the information that predicts who will survive, and for survival that information itself changes with age. Whether the lower operative rate in older patients reflects appropriate selection or age-based limitation of care cannot be resolved here and warrants dedicated study.

## Ethics approval and consent to participate

This study was approved by the Swedish Ethical Review Authority (DNR, 2025-02316-01). SweTrau data are collected under national registry regulations. The study was conducted in accordance with GDPR and Swedish data protection legislation. No individual patient contact was required.

## Author contributions

O.K: Conceptualization, Data curation, Formal analysis, Methodology, Writing – original draft.

N.M: Conceptualization, Methodology, Writing – review & editing.

Both authors: Approval of the final manuscript.

## Data availability

The data used in this study are derived from the Swedish Trauma Registry (SweTrau) and are not publicly available due to legal and ethical restrictions. Access requires approval from the registry and relevant authorities.

## Declaration of generative AI and AI-assisted technologies in the writing process

During the preparation of this manuscript, the authors used Claude (Anthropic) to support manuscript editing and formatting. After using this tool, the authors reviewed and edited the content as needed and take full responsibility for the content of the published article.

## Funding

No specific funding was received for this study.

## Declaration of competing interest

The authors declare that they have no known competing financial interests or personal relationships that could have appeared to influence the work reported in this paper.

## Figures and Tables

**Figure:**
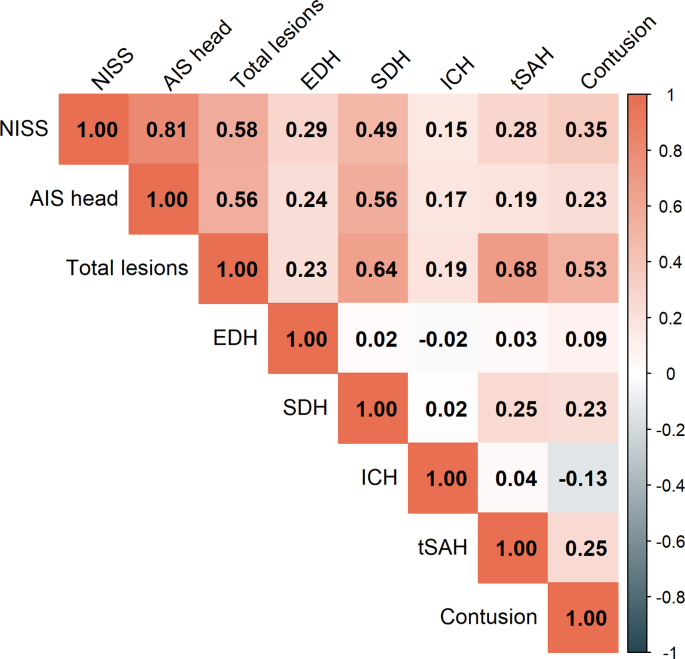
Supplementary Figure 1. Correlation matrix of injury-related variables Pairwise correlations between injury severity measures and intracranial lesion variables. Moderate clustering was observed among global injury severity measures (NISS, AIS head, and total lesion burden), while correlations between individual lesion types were generally low to moderate, indicating related but non-redundant information.

**Figure:**
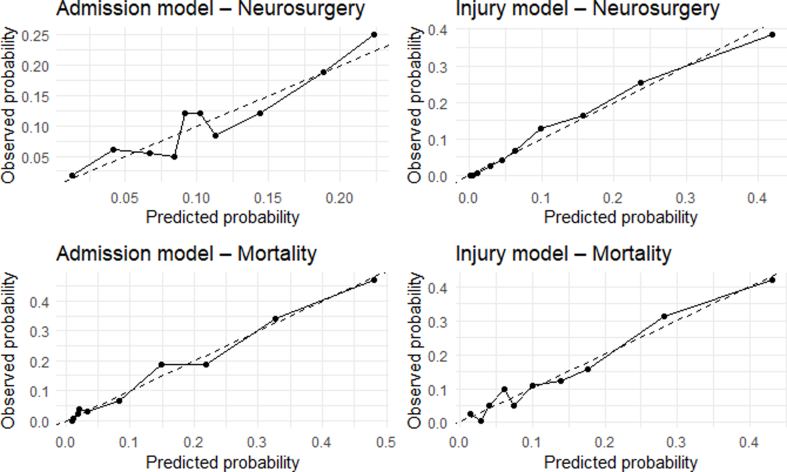
Supplementary Figure 2. Calibration plots. Calibration plots for admission- and injury-based models for neurosurgical intervention and in-hospital mortality. Calibration was assessed by comparing observed and predicted probabilities across deciles of predicted risk. The dashed line represents perfect calibration.

**Figure:**
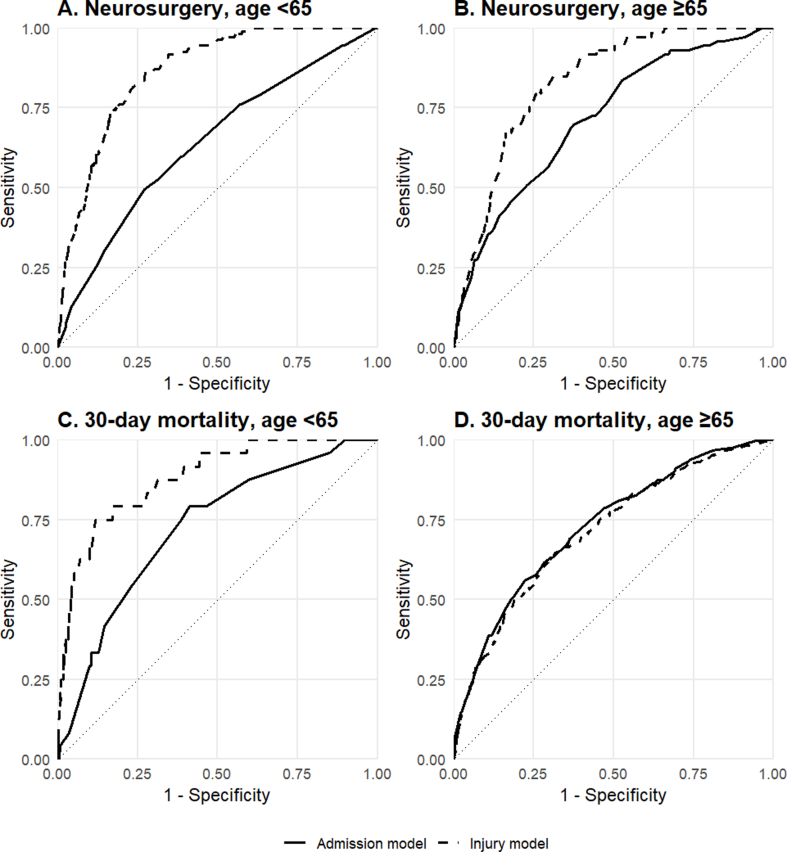
Supplementary Figure 3. Model discrimination by age stratum. Receiver operating characteristic curves for the admission model (solid) and the injury model (dashed) within each age stratum, for neurosurgical intervention (A, <65; B, ≥65) and 30-day mortality (C, <65; D, ≥65). For neurosurgical intervention the injury model discriminates better in both strata. For 30-day mortality the injury model is superior in patients <65 (C), whereas the two models are near-identical in those ≥65 (D). Corresponding areas under the curve and DeLong comparisons are given in Table 5. Curves are apparent estimates; in the smaller subgroups, particularly mortality in patients <65 (C), they indicate the direction of the difference rather than precise values.
