# Supplementary material for: Different predictors of neurosurgical intervention and mortality in moderate traumatic brain injury: a nationwide cohort study
Source: Brain Spine. 2026 Jul 14;6:106173. doi: 10.1016/j.bas.2026.106173 (PMC13427528; doi:10.1016/j.bas.2026.106173)
Supplement: Multimedia component 1 [file mmc1.docx]

Supplementary table 1: ICD-10 codes defining TBI

| **ICD-10 code** | **Description** |
| --- | --- |
| S06.0 | Concussion |
| S06.1 | Traumatic cerebral oedema |
| S06.2 | Diffuse brain injury |
| S06.3 | Focal brain injury |
| S06.4 | Epidural hemorrhage |
| S06.5 | Traumatic subdural hemorrhage |
| S06.6 | Traumatic subarachnoid hemorrhage |
| S06.7 | Intracranial injury with prolonged coma |
| S06.8 | Other intracranial injuries |
| S06.9 | Intracranial injury, unspecified |
| Supplementary Table 1. ICD-codes defining traumatic brain injury. | |

Supplementary table 2: Procedural codes

| **KVÅ code** | **Description** |
| --- | --- |
| AAD99 | Other operation for cranial or intracranial traumatic lesion |
| AAK10 | Duraplasty |
| AAA00 | Exploratory craniotomy |
| AWA00 | Reoperation for wound dehiscence after neurosurgical procedure |
| AAK85 | Reimplantation of previously removed skull bone (bone flap) |
| AAK50 | Reduction and fixation of skull fracture |
| AAD30 | Revision of penetrating or perforating skull injury |
| AAD05 | Evacuation of acute subdural hematoma |
| AAD00 | Evacuation of epidural hematoma |
| AAD10 | Evacuation of chronic subdural hematoma |
| AAD15 | Evacuation of traumatic intracerebral hematoma |
| AAA27 | Insertion of intracerebral pressure monitor |
| AAA20 | Insertion of intracranial pressure monitor |
| AA081 | Intracerebral pressure measurement |
| Supplementary Table 2. Swedish Classification of Health Interventions (Klassifikation av vårdåtgärder, KVÅ) used to define neurosurgical intervention. | |

Supplementary table 3: Collinearity diagnostics (GVIF) for variables included in multivariable models

| **Model** | **Variable** | **GVIF** | **Df** | **Adjusted GVIF** |
| --- | --- | --- | --- | --- |
| Mortality – Admission | Age group | 1.26 | 3 | 1.04 |
| Mortality – Admission | ASA class | 1.23 | 3 | 1.03 |
| Mortality – Admission | Sex | 1.05 | 1 | 1.02 |
| Mortality – Admission | GCS | 1.02 | 1 | 1.01 |
| Mortality – Admission | Hypotension | 1.01 | 1 | 1.01 |
| Mortality – Injury | tSAH | 3.3 | 1 | 1.82 |
| Mortality – Injury | NISS | 3.05 | 1 | 1.75 |
| Mortality – Injury | Total lesions | 7.82 | 2 | 1.67 |
| Mortality – Injury | SDH | 2.79 | 1 | 1.67 |
| Mortality – Injury | AIS head | 3.21 | 2 | 1.34 |
| Mortality – Injury | Contusion | 1.74 | 1 | 1.32 |
| Mortality – Injury | ICH | 1.51 | 1 | 1.23 |
| Mortality – Injury | EDH | 1.31 | 1 | 1.14 |
| Mortality – Injury | Polytrauma | 1.29 | 1 | 1.14 |
| Neurosurgery – Admission | Age group | 1.31 | 3 | 1.05 |
| Neurosurgery – Admission | ASA class | 1.3 | 3 | 1.04 |
| Neurosurgery – Admission | Sex | 1.02 | 1 | 1.01 |
| Neurosurgery – Admission | GCS | 1 | 1 | 1 |
| Neurosurgery – Admission | Hypotension | 1 | 1 | 1 |
| Neurosurgery – Injury | NISS | 2.21 | 1 | 1.49 |
| Neurosurgery – Injury | tSAH | 1.98 | 1 | 1.41 |
| Neurosurgery – Injury | Total lesions | 3.52 | 2 | 1.37 |
| Neurosurgery – Injury | Contusion | 1.67 | 1 | 1.29 |
| Neurosurgery – Injury | SDH | 1.59 | 1 | 1.26 |
| Neurosurgery – Injury | AIS head | 2.12 | 2 | 1.21 |
| Neurosurgery – Injury | EDH | 1.44 | 1 | 1.2 |
| Neurosurgery – Injury | ICH | 1.36 | 1 | 1.17 |
| Neurosurgery – Injury | Polytrauma | 1.24 | 1 | 1.11 |
| Supplementary Table 3. Assessment of multicollinearity in multivariable models  Variance inflation factors (GVIF), degrees of freedom (Df), and adjusted GVIF for all variables included in the multivariable admission and injury models for neurosurgical intervention and 30-day mortality. Adjusted GVIF values were low across all models, indicating no evidence of problematic multicollinearity. Abbreviations: AIS = Abbreviated Injury Scale; EDH = epidural hematoma; SDH = subdural hematoma; tSAH = traumatic subarachnoid hemorrhage; ICH = intracerebral hemorrhage. | | | | |

Supplementary table 4: Missing data for variables included in the analysis

| **Variable** | **N** | **Missing n (%)** | |
| --- | --- | --- | --- |
| Gender | 1761 | 0 | |
| Age | 1761 | 0 | |
| ASA | 1761 | 20 (1.1%) | |
| GCS | 1761 | 0 | |
| Hypotension | 1761 | 93 (5.3%) | |
| NISS | 1761 | 0 | |
| Polytrauma | 1761 | 0 | |
| AIS head | 1761 | 0 | |
| EDH | 1761 | 0 | |
| SDH | 1761 | 0 | |
| ICH | 1761 | 0 | |
| tSAH | 1761 | 0 | |
| Contusion | 1761 | 0 | |
| Total lesion count | 1761 | 0 | |
| Neurosurgical intervention | 1761 | 0 | |
| Mortality | 1761 | 0 | |
| Supplementary Table 4. Number and proportion of missing values for all variables included in the analysis. Missingness was low across all variables and not concentrated in key predictors. | | |  |

Supplementary table 5: Univariable logistic regression of variables associated with neurosurgical intervention

| **Variable** | **OR** | **95% CI** | **p-value** |
| --- | --- | --- | --- |
| **Sex** |  |  |  |
| F | — | — |  |
| M | 1.07 | 0.78, 1.47 | 0.7 |
| **Age** |  |  |  |
| 18-64 | — | — |  |
| 65-74 | 1.21 | 0.83, 1.74 | 0.3 |
| 75-84 | 0.62 | 0.39, 0.95 | 0.035* |
| 85+ | 0.20 | 0.08, 0.41 | <0.001* |
| **ASA class** |  |  |  |
| ASA 1 | — | — |  |
| ASA 2 | 1.08 | 0.75, 1.55 | 0.7 |
| ASA 3 | 0.86 | 0.59, 1.26 | 0.4 |
| ASA 4+ | 0.21 | 0.01, 1.00 | 0.13 |
| **GCS** |  |  |  |
| 9-11 | — | — |  |
| 12-13 | 0.50 | 0.37, 0.67 | <0.001* |
| **Hypotension** |  |  |  |
| No | — | — |  |
| Yes | 0.83 | 0.20, 2.36 | 0.8 |
| **NISS** | 1.08 | 1.07, 1.09 | <0.001* |
| **Polytrauma** |  |  |  |
| No | — | — |  |
| Yes | 1.54 | 1.06, 2.20 | 0.021* |
| **AIS head** |  |  |  |
| 0-2 | — | — |  |
| 3-4 | 24.4 | 7.65, 149 | <0.001* |
| 5+ | 123 | 38.7, 750 | <0.001* |
| **EDH** |  |  |  |
| No | — | — |  |
| Yes | 4.64 | 3.17, 6.74 | <0.001* |
| **SDH** |  |  |  |
| No | — | — |  |
| Yes | 3.67 | 2.64, 5.18 | <0.001* |
| **ICH** |  |  |  |
| No | — | — |  |
| Yes | 1.47 | 0.88, 2.35 | 0.13 |
| **Traumatic SAH** |  |  |  |
| No | — | — |  |
| Yes | 1.56 | 1.15, 2.12 | 0.004* |
| **Contusion** |  |  |  |
| No | — | — |  |
| Yes | 2.26 | 1.67, 3.04 | <0.001* |
| **Total lesions** |  |  |  |
| 0 | — | — |  |
| 1 | 6.00 | 2.74, 15.8 | <0.001* |
| 2+ | 14.3 | 6.84, 36.7 | <0.001* |
| Supplementary table 5. Univariable logistic regression of variables associated with neurosurgical intervention  Abbreviations: OR = odds ratio; CI = confidence interval; NISS = New Injury Severity Score; AIS = Abbreviated Injury Scale; EDH = epidural hematoma; SDH = subdural hematoma; tSAH = traumatic subarachnoid hemorrhage; ICH = intracerebral hemorrhage. Statistically significant values (p < 0.05) are marked with an asterisk. | | | |

Supplementary table 6: Univariable logistic regression of variables associated with 30-day mortality

| **Variable** | **OR** | **95% CI** | **p-value** |
| --- | --- | --- | --- |
| **Sex** |  |  |  |
| F | — | — |  |
| M | 0.65 | 0.50, 0.83 | <0.001* |
| **Age** |  |  |  |
| 18-64 | — | — |  |
| 65-74 | 6.36 | 3.89, 10.6 | <0.001* |
| 75-84 | 16.5 | 10.6, 26.6 | <0.001* |
| 85+ | 38.5 | 24.6, 62.5 | <0.001* |
| **ASA class** |  |  |  |
| ASA 1 | — | — |  |
| ASA 2 | 4.15 | 2.52, 7.17 | <0.001* |
| ASA 3 | 12.7 | 7.95, 21.3 | <0.001* |
| ASA 4+ | 46.0 | 20.9, 106 | <0.001* |
| **GCS** |  |  |  |
| 9-11 | — | — |  |
| 12-13 | 0.60 | 0.47, 0.78 | <0.001* |
| **Hypotension** |  |  |  |
| No | — | — |  |
| Yes | 1.55 | 0.65, 3.32 | 0.3 |
| **NISS** | 1.04 | 1.03, 1.05 | <0.001* |
| **Polytrauma** |  |  |  |
| No | — | — |  |
| Yes | 0.97 | 0.69, 1.36 | 0.9 |
| **AIS head** |  |  |  |
| 0-2 | — | — |  |
| 3-4 | 2.65 | 1.79, 4.04 | <0.001* |
| 5+ | 9.28 | 6.16, 14.4 | <0.001* |
| **EDH** |  |  |  |
| No | — | — |  |
| Yes | 0.74 | 0.45, 1.17 | 0.2 |
| **SDH** |  |  |  |
| No | — | — |  |
| Yes | 2.69 | 2.07, 3.52 | <0.001* |
| **ICH** |  |  |  |
| No | — | — |  |
| Yes | 1.90 | 1.26, 2.81 | 0.002* |
| **Traumatic SAH** |  |  |  |
| No | — | — |  |
| Yes | 2.04 | 1.57, 2.65 | <0.001* |
| **Contusion** |  |  |  |
| No | — | — |  |
| Yes | 0.79 | 0.60, 1.04 | 0.10 |
| **Total lesions** |  |  |  |
| 0 | — | — |  |
| 1 | 2.81 | 1.83, 4.45 | <0.001* |
| 2+ | 3.63 | 2.43, 5.62 | <0.001* |
| Supplementary table 6. Univariable logistic regression of variables associated with 30-day mortality. Abbreviations: OR = odds ratio; CI = confidence interval; NISS = New Injury Severity Score; AIS = Abbreviated Injury Scale; EDH = epidural hematoma; SDH = subdural hematoma; tSAH = traumatic subarachnoid hemorrhage; ICH = intracerebral hemorrhage. Statistically significant values (p < 0.05) are marked with an asterisk. | | | |

# Figures

Figure 1. Study flowchart

Flowchart of patient selection from the Swedish Trauma Registry (SweTrau) between 2018 and 2023. Patients aged ≥18 years with traumatic brain injury and available emergency department Glasgow Coma Scale (GCS) scores were included. Moderate traumatic brain injury was defined as GCS 9–13.

Abbreviations: TBI = traumatic brain injury; GCS = Glasgow Coma Scale; ED = emergency department.

Figure 2. Receiver operating characteristic curves for prediction models

Receiver operating characteristic curves for logistic regression models predicting neurosurgical intervention (A) and 30-day mortality (B) based on admission variables and injury severity variables.

Abbreviations: ROC = receiver operating characteristic; AUC = area under the receiver operating characteristic curve.

Figure 3. Forest plots of multivariable admission models

Forest plots showing adjusted odds ratios with 95% confidence intervals from multivariable logistic regression models. Panels A and B show results from the admission model for neurosurgical intervention (A) and 30-day mortality (B). Panels C and D show results from the injury model for neurosurgical intervention (C) and 30-day mortality (D).

Abbreviations: OR = odds ratio; CI = confidence interval; ASA = American Society of Anesthesiologists; GCS = Glasgow Coma Scale; NISS = New Injury Severity Score; AIS = Abbreviated Injury Scale; EDH = epidural hematoma; SDH = subdural hematoma; tSAH = traumatic subarachnoid hemorrhage; ICH = intracerebral hemorrhage.

Figure 4. Diverging associations between age and outcomes

Adjusted odds ratios for age group from the multivariable admission models, with 18–64 years as the reference. Circles show neurosurgical intervention; triangles show 30-day mortality. With increasing age, the adjusted odds of death rise while the adjusted odds of neurosurgical intervention fall, the two associations diverging across age groups. Error bars are 95% confidence intervals; the odds-ratio axis is on a logarithmic scale.

Abbreviations: OR = odds ratio; CI = confidence interval.

Supplementary Figure 1. Correlation matrix of injury-related variables

Pairwise correlations between injury severity measures and intracranial lesion variables. Moderate clustering was observed among global injury severity measures (NISS, AIS head, and total lesion burden), while correlations between individual lesion types were generally low to moderate, indicating related but non-redundant information.

Supplementary Figure 2. Calibration plots.

Calibration plots for admission- and injury-based models for neurosurgical intervention and in-hospital mortality. Calibration was assessed by comparing observed and predicted probabilities across deciles of predicted risk. The dashed line represents perfect calibration.

**Supplementary Figure 3.** **Model discrimination by age stratum***.* Receiver operating characteristic curves for the admission model (solid) and the injury model (dashed) within each age stratum, for neurosurgical intervention (A, <65; B, ≥65) and 30-day mortality (C, <65; D, ≥65). For neurosurgical intervention the injury model discriminates better in both strata. For 30-day mortality the injury model is superior in patients <65 (C), whereas the two models are near-identical in those ≥65 (D). Corresponding areas under the curve and DeLong comparisons are given in Table 5. Curves are apparent estimates; in the smaller subgroups, particularly mortality in patients <65 (C), they indicate the direction of the difference rather than precise values.
